# Association of Viral Persistence and Atherosclerosis in Adults With Treated HIV Infection

**DOI:** 10.1001/jamanetworkopen.2020.18099

**Published:** 2020-10-29

**Authors:** Megan M. McLaughlin, Yifei Ma, Rebecca Scherzer, Smruti Rahalkar, Jeffrey N. Martin, Claire Mills, Jeffrey Milush, Steven G. Deeks, Priscilla Y. Hsue

**Affiliations:** 1 Department of Medicine, University of California, San Francisco (UCSF); 2 Department of Medicine, San Francisco Veterans Affairs Medical Center, UCSF; 3 Division of Cardiology, Department of Medicine, San Francisco General Hospital, UCSF; 4 Department of Epidemiology and Biostatistics, UCSF; 5 Department of Medicine, Division of Experimental Medicine, UCSF; 6 Positive Health Program, San Francisco General Hospital, San Francisco, California

## Abstract

**Importance:**

Persons living with HIV (PLWH) have increased risk for cardiovascular disease, and inflammation is thought to contribute to this excess risk. Production of HIV during otherwise effective antiretroviral therapy (ART) has been associated with inflammation.

**Objective:**

To determine whether higher levels of viral persistence are associated with atherosclerosis as assessed by changes in carotid artery intima-media thickness (IMT) over time.

**Design, Setting, and Participants:**

In this cohort study, intima-media thickness, a validated marker of atherosclerosis, was assessed over time in a cohort of treated PLWH with viral suppression. Cell-associated HIV DNA and RNA and change in IMT, adjusted for demographics, cardiovascular risk factors, and HIV-related factors, were examined, as well as which factors were associated with viral persistence. One hundred fifty-two PLWH with undetectable viral loads for at least 6 months before study enrollment were recruited from HIV clinics affiliated with 2 hospitals in San Francisco, California, from January 1, 2003, to December 31, 2012. Data were analyzed from February 7, 2018, to May 12, 2020.

**Exposures:**

Cell-associated HIV RNA and DNA were measured using enriched CD4^+^ T cells from cryopreserved peripheral blood mononuclear cells.

**Main Outcomes and Measures:**

Carotid IMT was measured at baseline and the last visit, with a mean (SD) follow-up of 4.2 (2.7) years, using high-resolution B mode ultrasonography. The main study outcomes were baseline IMT, annual IMT progression, and incident plaque, defined as a focal region of carotid IMT of greater than 1.5 mm.

**Results:**

The analysis included 152 PLWH (140 [92.1%] male; median age, 48.5 [interquartile range {IQR}, 43.3-53.7] years). Older age, smoking, medications for hypertension, higher low-density lipoprotein levels, and higher interleukin 6 levels were associated with higher baseline mean IMT, whereas cell-associated HIV DNA (estimate, −0.07% [95% CI, −6.1% to 6.4%]; *P* = .98), and HIV RNA levels (estimate, −0.8% [95% CI, −5.9% to 4.4%]; *P* = .75) were not. Levels of HIV RNA (0.017 [95% CI, 0.000-0.034] mm/y; *P* = .047) and HIV DNA (0.022 [95% CI, 0.001-0.044] mm/y; *P* = .042) were significantly associated with annual carotid artery IMT progression in unadjusted models only. Both HIV RNA (incidence risk ratio [IRR], 3.05 [95% CI, 1.49-6.27] per IQR; *P* = .002) and HIV DNA (IRR, 3.15 [95% CI, 1.51-6.57] per IQR; *P* = .002) were significantly associated with incident plaque, which remained significant after adjusting for demographics, cardiovascular risk factors, and HIV-related factors (IRR for HIV RNA, 4.05 [95% CI, 1.44-11.36] per IQR [*P* = .008]; IRR for HIV DNA, 3.35 [95% CI, 1.22-9.19] per IQR [*P* = .02]). Higher C-reactive protein levels were associated with higher cell-associated HIV RNA (estimate, 20.7% [95% CI, 0.9%-44.4%] per doubling; *P* = .04), whereas higher soluble CD14 levels were associated with HIV DNA (estimate, 18.6% [95% CI, 3.5%-35.8%] per 10% increase; *P* = .01). Higher soluble CD163 levels were associated with a higher HIV RNA:DNA ratio (difference, 63.8% [95% CI, 3.5%-159.4%]; *P* = .04).

**Conclusions and Relevance:**

These findings suggest that measurements of viral persistence in treated HIV disease are independently associated with incident carotid plaque development. The size and transcriptional activity of the HIV reservoir may be important contributors to HIV-associated atherosclerosis.

## Key Points

**Question:**

Are higher levels of HIV persistence associated with atherosclerosis as assessed by changes in carotid artery intima-media thickness (IMT) progression over time?

**Findings:**

In this cohort study of 152 adults living with HIV and with suppressed viral loads, higher levels of both cell-associated HIV RNA and HIV DNA were associated with incident plaque development but not baseline IMT or annual IMT progression.

**Meaning:**

These findings suggest that the size and transcriptional activity of the HIV reservoir may be important contributors to HIV-associated atherosclerosis.

## Teaser

This cohort study assesses whether higher levels of viral persistence are associated with atherosclerosis as assessed by changes in carotid artery intima-media thickness over time in patients living with HIV.

## Introduction

The global burden of cardiovascular disease (CVD) in HIV has tripled during the past 2 decades,^1^ and the risk of both CVD and myocardial infarction is increased 2-fold in persons living with HIV (PLWH).^2,3^ This excess risk is owing in part to a higher prevalence of traditional risk factors in PLWH, but HIV-associated inflammation is likely also a key contributor. Other studies^3,4,5^ have established that uncontrolled HIV disease is associated with increased cardiovascular risk. However, higher rates of myocardial infarction and atherosclerosis are still present in the setting of treated and suppressed HIV disease. Despite control of viremia with antiretroviral therapy (ART), inflammation persists at levels higher than in the uninfected person.^6^ In turn, inflammatory and coagulation markers are strongly associated with cardiovascular events, mortality, and increased cardiovascular risk in a variety of HIV-infected populations.^7,8,9,10^

Imaging studies suggest that the type of atherosclerosis in PLWH may be distinct from that in the general population. Namely, PLWH have higher arterial inflammation compared with uninfected controls,^11^ and noncalcified plaque is more common in PLWH.^12,13^ Carotid artery intima-media thickness (IMT) is a direct evaluation of the arterial wall^14^ and accordingly has the potential to provide mechanistic insight into the pathogenesis of HIV-associated atherosclerosis. It is also a validated marker of atherosclerosis that has been shown to be associated with CVD events.^15,16^

As a retrovirus, HIV integrates its genome into the host DNA. Any infected cells can consequently initiate new rounds of infection. Antiretroviral therapy prevents HIV from spreading and infecting new cells but does not eliminate those cells already infected. Because memory CD4^+^ T cells are maintained indefinitely via homeostatic and antigen-specific cell proliferation,^17,18,19^ the virus persists even during long-term ART. The size of this viral reservoir can be estimated by measuring the frequency of HIV DNA in circulating memory CD4^+^ T cells.^20^ The degree to which this reservoir is potentially “active” and produces new viral products or virions can be estimated by measuring the levels of HIV RNA in circulating cells.^21,22^ Notably, much of the HIV DNA is defective and cannot support the production of replication-competent virions, but it can support the production of viral proteins.^23,24^ The amount of cell-associated DNA and RNA in blood has been associated with levels of T-cell activation, T-cell proliferation, and soluble markers of inflammation in many^25,26,27,28,29^ but not all^30^ studies.

Given the potential role of HIV persistence in driving inflammation and given the role of inflammation in driving the development of coronary artery disease, we hypothesized that HIV DNA and RNA levels would be associated with overall atherosclerosis burden. We therefore examined the association between viral persistence and baseline carotid IMT, annual IMT progression, and incident plaque in a cohort of treated PLWH with viral suppression, controlling for traditional cardiovascular risk factors, HIV-related factors, and inflammatory markers. We also measured the association between inflammatory markers and HIV DNA and RNA levels.

## Methods

### Study Population

We conducted a longitudinal study of PLWH, who at our baseline visit were receiving ART and had an undetectable viral load. All participants were initially enrolled in the University of California, San Francisco–based SCOPE (Study of the Consequences of the Protease Inhibitor Era) cohort,^31^ a longitudinal observational cohort of PLWH recruited from HIV/AIDS clinics at the San Francisco General Hospital and the San Francisco Veterans Affairs Medical Center from January 1, 2003, to December 31, 2012. From this cohort, we invited all participants to enroll in a substudy in which IMT was measured, as previously described.^4,32,33,34^ For the present analysis, we selected those individuals who were receiving ART and who at their first IMT visit had an undetectable viral load for at least the past 6 months. Participants were not recruited for any of these studies based on cardiovascular risk. Candidate covariates were measured at the same time as the baseline IMT. Participants in the SCOPE cohort underwent blood tests and filled out questionnaires regarding medications, health-related behaviors, and symptoms every 4 months. Clinical data, including ART history, were obtained by extensive medical record review. The University of California, San Francisco, Committee on Human Research approved the study, and all participants provided written informed consent. This study followed the Strengthening the Reporting of Observational Studies in Epidemiology (STROBE) reporting guideline.

### Carotid IMT Measurement

Carotid IMT was measured at baseline and the last visit, which occurred during a mean (SD) follow-up of 4.2 (2.7) years. High-resolution B mode ultrasonography was used to measure carotid IMT in the near and far walls of the bilateral common carotid, bifurcation, and internal carotid regions, according to the standardized protocol of the Atherosclerosis Risk in Communities Study, as previously described.^4,35^ Within each segment (common, bifurcation, and internal), we calculated IMT as the mean of the near and far walls of the bilateral carotid arteries, blinded to other laboratory values. Overall mean carotid IMT was calculated as the mean of the 12 segments. Plaque was defined as a focal region of carotid IMT greater than 1.5 mm. Incident plaque was defined as no plaque at baseline visit and plaque at last visit. Progression of IMT was defined as any positive change in carotid IMT from baseline to last visit. Annual IMT progression was defined as continuous absolute change in carotid IMT from baseline visit to last visit divided by the time between baseline and the last visit.

### Measurement of Viral Persistence and Plasma Biomarkers

Viral persistence parameters were measured at baseline only. Using thawed, cryopreserved peripheral blood mononuclear cells, total cellular RNA and DNA from enriched CD4^+^ T cells were purified and measured using quantified polymerase chain reaction analysis. Detailed laboratory methods are described in the eMethods in the Supplement. Soluble markers of inflammation (levels of interleukin 6 [IL-6], tumor necrosis factor, C-reactive protein, soluble CD14 [sCD14], and sCD163) and coagulopathy (levels of D-dimer) were measured at baseline in cryopreserved plasma samples using a multiplex electrochemiluminescence assay (Meso Scale Discovery).

### Statistical Analysis

Data were analyzed from February 7, 2018, to May 12, 2020. For our primary analyses examining carotid IMT as the outcome, candidate covariates were assessed at baseline and included age, sex, race/ethnicity, cigarette smoking, diabetes, hypertension, history of CVD, low-density lipoprotein cholesterol levels, body mass index, use of medication to lower cholesterol levels, use of antihypertensives, duration of HIV infection, duration of ART use, CD4 T-cell count or CD4:CD8 T-cell ratio, hepatitis C virus coinfection, and plasma IL-6 level.

We used generalized linear models to examine the bivariable associations between candidate covariates and (1) baseline carotid IMT and (2) annual IMT progression. We then used a staged multivariable approach to examine the association between each of the 3 primary independent variables (HIV DNA, HIV RNA, and RNA:DNA ratio) and the 2 outcomes (1) alone, (2) adjusted for demographic factors, and (3) adjusted for demographic factors plus traditional cardiovascular and HIV-related risk factors. In addition to the full multivariable models, we also examined models adjusted for demographic factors plus 1 additional cardiovascular or HIV-related risk factor. HIV DNA and RNA are known to have extremely right-skewed distribution. In these models, we normalized them by dividing by their interquartile ranges (IQRs) (on a log scale), so their coefficients reflect change from the 25th to the 75th percentile. In the models in which baseline carotid IMT and HIV DNA, HIV RNA, and RNA:DNA ratio are dependent variables, we log-transformed them to stabilize the residual variance to best fit the models and then exponentiated the coefficients to present estimated percentage differences.

We used Poisson regression models with log-transformed time of exposure as an offset to examine the bivariable associations between candidate covariates and the outcome of incident plaque among individuals who did not have plaque at baseline.^36,37^ A robust variance estimator was applied to adjust for potential overdispersion in the models. We then used a staged multivariable approach, as described above, to examine the association of each of the 3 primary independent variables (HIV DNA, HIV RNA, and RNA:DNA ratio) with incident plaque. Incidence risk ratios (IRRs) were calculated for all of these models.

Because CD4:CD8 ratio and CD4^+^ T-cell counts are associated with the measures of viral persistence,^29,38^ we performed sensitivity analyses in which we removed CD4:CD8 ratio or CD4 count from each of the models examining incident baseline carotid IMT, annual IMT progression, and incident plaque as outcomes. For our secondary analyses, we sought to identify factors associated with HIV RNA, HIV DNA, and RNA:DNA ratio. We created models that were adjusted for demographics and additionally included HIV-related factors and markers of inflammation (levels of IL-6, tumor necrosis factor, C-reactive protein, sCD14, and sCD163) or coagulopathy (level of D-dimer) that remained significant in the multivariable models.

In linear regression models with randomly missing inflammatory markers, we used the full-information maximum-likelihood approach in the setting of path analysis over multiple imputation approach for its efficiency.^39^ In the Poisson analyses, however, multiple imputation with the Markov chain Monte Carlo method was used, with the number of repetitions similar to a percentage of missing values.^40,41^ The variables used to create the imputation model included demographics and the cardiovascular and HIV-related risk factors specified above. Multiple imputation estimates of model parameters were computed by calculating the mean estimates from imputed models, and the variance and 95% CI of these estimates were computed using Rubin’s combining formula.^42^

All analyses were conducted using the SAS system, version 9.4 (SAS Institute Inc). Two-sided *P* < .05 indicated significance.

## Results

### Baseline Characteristics

As shown in Table 1, among the 152 participants, 107 were White (70.4%), 140 were male (92.1%), and 12 were female (8.0%), with a median age of 48.5 (IQR, 43.3-53.7) years. The median duration of HIV infection was 13 (IQR, 10-17) years, and the median CD4 cell count was 461.5 (IQR, 309.0-643.5). Individuals were treated with ART for a median duration of 5.3 (IQR, 3.8-7.5) years. Traditional risk factors were common and included 91 (59.9%) ever smokers, 45 (29.6%) with hypertension, and 34 (22.4%) with a family history of premature CVD. The median 10-year atherosclerotic CVD risk of the study participants was 5.3% (IQR, 3.1%-9.3%).

**Table 1.** Summary of Baseline Demographic and Clinical Characteristics of the Cohort

Characteristic	Data (n = 152)^a^
Demographic factors	
Age, median (IQR), y	48.5 (43.3-53.7)
Race/ethnicity, No. (%)	
White	107 (70.4)
Black	24 (15.8)
Latino	12 (7.9)
Other	9 (5.9)
Sex, No. (%)	
Male	140 (92.1)
Female	12 (8.0)
Clinical factors	
BMI, median (IQR)	24.4 (22.6-27.9)
Systolic blood pressure, median (IQR), mm Hg	122.5 (115.0-131.0)
Diastolic blood pressure, median (IQR), mm Hg	76.0 (68.5-82.0)
Use of medication to lower cholesterol level, No. (%)	42 (27.6)
Use of antihypertensives, No. (%)	37 (24.3)
Baseline coronary artery disease or cerebrovascular accident	10 (6.6)
Use of aspirin therapy	43 (28.4)
10-y Atherosclerotic cardiovascular disease risk, median (IQR), %	5.3 (3.1-9.3)
HIV-related risk factors	
HIV duration, median (IQR), y	13 (10-17)
CD4 count, median (IQR)	461.5 (309.0-643.5)
CD4 count nadir, median (IQR)	99.0 (20.0-197.5)
CD4:CD8 ratio, median (IQR)	0.4 (0.3-0.8)
Duration of nonnucleoside reverse-transcriptase inhibitor use, median (IQR), y	2.2 (0-4.2)
Duration of nucleoside reverse-transcriptase inhibitor use, median (IQR), y	7.3 (4.6-10.6)
Duration of abacavir use, median (IQR), y	0.0 (0.0-1.0)
Duration of protease inhibitor use, median (IQR), y	4.5 (2.3-7.0)
Duration of ART use, median (IQR), y	5.3 (3.8-7.5)
Opportunistic infection, No. (%)	78 (51.3)
Comorbidities	
Diabetes, No. (%)^b^	11 (7.2)
Hepatitis C virus coinfection, No. (%)	28 (18.4)
Family history of premature CVD, No. (%)	34 (22.4)
Hypertension, No. (%)^c^	45 (29.6)
Ever smoker, No. (%)	91 (59.9)
Smoking, median (IQR), pack-years	4 (0-19)
Cocaine use, No. (%)	9 (5.9)
Methamphetamine use, No. (%)	13 (8.6)
Laboratory values, median (IQR)	
Total cholesterol level, mg/dL	191 (169.5-220.0)
LDL-C level, mg/dL	105 (86.0-129.5)
HDL-C level, mg/dL	44 (35-53)
Triglyceride level, mg/dL	162 (96-318)
Inflammatory markers, median (IQR)	
C-reactive protein level, mg/L	1.8 (0.7-4.5)
Soluble CD14 level, μg/mL	1.9 (1.6-2.2)
IL-6 level, pg/mL	1.1 (0.7-1.9)
Soluble CD163 level, ng/mL	475.0 (330.4-645.4)
D-dimer level, mg/L	0.33 (0.22-0.50)
Viral persistence, median (IQR)	
HIV RNA level, log^10^ copies/10^6^ CD4^+^ T cells	2158.3 (545.8-7444.6)
HIV DNA level, log^10^ copies/10^6^ CD4^+^ T cells	816.0 (98.1-3423.1)
HIV RNA:DNA ratio	2.6 (1.2-12.3)

Abbreviations: ART, antiretroviral therapy; BMI, body mass index (calculated as weight in kilograms divided by square of height in meters); CVD, cardiovascular disease; HDL-C, high-density lipoprotein; IL, interleukin; IQR, interquartile range; LDL-C, low-density lipoprotein cholesterol.

SI conversion factors: To convert cholesterol to mmol/L, multiply by 0.0259; C-reactive protein to mg/L, multiply by 10; D-dimer to nmol/L, multiply by 5.476; and triglycerides to mmol/L, multiply by 0.0113.

^a^ Among 152 study participants, 14 are missing CD4:CD8 ratio; 1, ART use; 16, LDL-C; 33, soluble CD14, IL-6, and soluble CD163; and 3, RNA and RNA:DNA ratio. Percentages have been rounded and may not total 100.

^b^ Ascertained by medical record review. Patients who had a diagnosis of diabetes (type 1 or 2) were included.

^c^ Ascertained by 2 consecutive blood pressure readings with systolic blood pressure greater than 140 mm Hg or diastolic blood pressure greater than 90 mm Hg.

The median cell-associated HIV RNA level was 2158.3 (IQR, 545.8-7444.6) copies/10^6^ CD4^+^ T cells. The median HIV DNA level was 816.0 (IQR, 98.1-3423.1) copies/10^6^ CD4^+^ T cells. The median RNA:DNA ratio was 2.6 (IQR, 1.2-12.3).

The mean (SD) duration of follow-up was 4.2 (2.7) years. Mean (SD) carotid IMT at baseline was 1.2 (0.3) mm. The mean (SD) IMT at last visit was 1.23 (0.40) mm, and the mean (SD) IMT progression was 0.08 (0.90) mm/y. At baseline, 86 of 152 individuals (56.6%) had plaque. Eighty-two of these 86 individuals (95.3%) who had plaque at baseline had IMT progression during the study period. Of the 66 individuals without plaque at baseline, 21 (31.8%) had incident plaque during the study period.

### Traditional Risk Factors Associated With Baseline IMT

We first evaluated factors associated with higher baseline carotid IMT. Cell-associated HIV RNA, HIV DNA, and the RNA:DNA ratio were not significantly associated with baseline carotid IMT in any of the models examined (Table 2). In our multivariable models, older age (difference, 16.6% [95% CI, 10.2%-23.4%; *P* < .001] for the model including HIV DNA and 16.5% [95% CI, 10.1%-23.2%; *P* < .001] for the model including HIV RNA), smoking (difference, 3.4% [95% CI, 0.7%-6.3%; *P* = .01] for the model including HIV DNA and 3.4% [0.7%-6.2%; *P* = .01] for the model including HIV RNA), medications for hypertension (difference, 19.5% [95% CI, 1.4%-40.9%; *P* = .03] for the model including HIV DNA and 19.3% [95% CI, 1.2%-40.6%; *P* = .04] for the model including HIV RNA), higher low-density lipoprotein levels (difference, 15.2% [95% CI, 6.2%-25.0%; *P* < .001] for the model including HIV DNA and 15.3% [95% CI, 6.3%-25.2%; *P* < .001] for the model including HIV RNA), and higher IL-6 levels (difference, 4.9% [95% CI, 0.5%-9.4%; *P* = .03] for the model including HIV DNA and 5.0% [95% CI, 0.6%-9.5%; *P* = .02] for the model including HIV RNA) were associated with greater carotid IMT at baseline. Removing the CD4:CD8 ratio from the models did not affect the association between the measures of viral persistence and baseline carotid IMT.

**Table 2.** Association of Viral Persistence Measures With Relative Differences in Baseline Mean Carotid IMT^a^

Parameter	Unadjusted		Demographic adjusted^b^		Multivariable adjusted^c^	
	Estimate, % (95% CI)	*P* value	Estimate, % (95% CI)	*P* value	Estimate, % (95% CI)	*P* value
RNA (per IQR)	3.2 (−2.7 to 9.4)	.30	−0.5 (−5.5 to 4.8)	.86	−0.8 (−5.9 to 4.4)	.75
DNA (per IQR)	2.1 (−5.2 to 9.9)	.59	0.9 (−5.3 to 7.5)	.78	−0.07 (−6.1 to 6.4)	.98
RNA:DNA ratio, tertile 2 vs 1	2.0 (−9.1 to 14.4)	.74	2.0 (−7.7 to 12.6)	.70	5.1 (−4.4 to 15.5)	.31
RNA:DNA, ratio tertile 3 vs 1	−2.1 (−12.7 to 9.9)	.72	1.3 (−8.3 to 11.8)	.80	3.8 (−5.5 to 14.1)	.44

Abbreviations: IMT, intima-media thickness; IQR, interquartile range.

^a^ Includes 152 participants. A full-information maximum-likelihood approach was used for missing values.

^b^ Controlled for age, sex, and race.

^c^ Controlled for demographics (age, sex, and race), traditional cardiovascular risk factors (smoking, diabetes, hypertension, history of cardiovascular disease, low-density lipoprotein level, body mass index, use of medication to lower cholesterol level, and use of antihypertensives), HIV-related risk factors (duration of HIV infection, duration of use of antiretroviral therapy, and CD4:CD8 ratio), hepatitis C virus coinfection, and interleukin 6 level.

### Assessment of Factors Associated With IMT Progression

We then determined which factors were associated with greater annual carotid IMT progression during the study period. Older age (estimate, 0.031 [95% CI, 0.012-0.050; *P* = .002] for the model including HIV DNA; 0.030 [95% CI, 0.011-0.049; *P* = .002] for the model including HIV RNA), male sex (estimate, −0.069 [95% CI, −0.046 to 0.030; *P* = .02] for the model including HIV DNA; −0.065 [95% CI, −0.119 to −0.011; *P* = .02] for the model including HIV RNA), hypertension (estimate, 0.062 [95% CI, 0.011-0.113; *P* = .02] for the model including HIV DNA; 0.061 [95% CI, 0.010-0.112; *P* = .02] for the model including HIV RNA), and use of antihypertensives (estimate, −0.077 [95% CI, −0.132 to −0.021; *P* = .007] for the model including HIV DNA; −0.077 [95% CI, −0.132 to −0.022; *P* = .006] for the model including HIV RNA) were consistently associated with greater IMT progression in multivariable models. In the unadjusted models, both cell-associated HIV RNA (0.017 [95% CI, 0.001-0.034] mm/y; *P* = .047) and HIV DNA (0.022 [0.001-0.044] mm/y; *P* = .042) were associated with greater annual IMT progression (Table 3). However, this association did not remain significant in the demographic-adjusted and multivariable models (Table 3). The HIV RNA:DNA ratio was significant in the demographic-adjusted model, with HIV RNA:DNA tertile 2 being associated with decreased carotid IMT progression (−0.032 [95% CI, −0.063 to −0.001] mm/y; *P* = .048) compared with tertile 1, but this association did not remain significant in the full multivariable model. After removing the CD4:CD8 ratio, RNA:DNA ratio remained significantly associated with decreased carotid IMT progression for tertile 2 vs 1 (−0.032 [95% CI, −0.063 to −0.001] mm/y; *P* = .045) and tertile 3 vs 1 (−0.032 [95% CI, −0.063 to −0.001]; *P* = .04) (eTables 1 and 2 in the Supplement), but the other models did not change significantly.

**Table 3.** Association of Viral Persistence Measures With Annual Carotid IMT Progression^a^

Parameter	Unadjusted		Demographic adjusted^b^		Multivariable adjusted^c^	
	Annual difference in carotid IMT (95% CI), mm	*P* value	Annual difference in carotid IMT (95% CI), mm	*P* value	Difference in carotid IMT (95% CI), mm	*P* value
RNA (per IQR)	0.017 (0.000 to 0.034)	.047	0.009 (−0.007 to 0.026)	.28	0.001 (−0.016 to 0.019)	.87
DNA (per IQR)	0.022 (0.001 to 0.044)	.042	0.016 (−0.005 to 0.037)	.13	0.011 (−0.010 to 0.032)	.29
RNA:DNA ratio, tertile 2 vs 1	−0.029 (−0.063 to 0.004)	.09	−0.032 (−0.063 to −0.001)	.048	−0.029 (−0.061 to 0.002)	.07
RNA:DNA ratio, tertile 3 vs 1	−0.033 (−0.066 to 0.001)	.06	−0.025 (−0.057 to 0.007)	.12	−0.030 (−0.061 to 0.002)	.06

Abbreviations: IMT, intima-media thickness; IQR, interquartile range.

^a^ Includes 152 participants. A full-information maximum-likelihood approach was used for missing values.

^b^ Controlled for age, sex, and race.

^c^ Controlled for demographics (age, sex, and race), traditional cardiovascular risk factors (smoking, diabetes, hypertension, history of cardiovascular disease, low-density lipoprotein level, body mass index, use of medication to lower cholesterol level, and use of antihypertensives), HIV-related risk factors (duration of HIV infection, duration of use of antiretroviral therapy, and CD4:CD8 ratio), hepatitis C virus coinfection, and interleukin 6 level.

### Independent Association of HIV RNA and DNA With Incident Plaque

Among individuals who did not have plaque at baseline (Table 4), both HIV RNA (IRR, 3.05 [95% CI, 1.49-6.27] per IQR; *P* = .002) and HIV DNA (IRR, 3.15 [95% CI, 1.51-6.57] per IQR; *P* = .002) were associated with higher incident plaque development in unadjusted models. These associations also persisted and remained highly significant in multivariable-adjusted models for HIV RNA (IRR, 4.05 [95% CI, 1.44-11.36] per IQR; *P* = .008) and HIV DNA (IRR, 3.35 [95% CI, 1.22-9.19] per IQR; *P* = .02). In contrast, HIV RNA:DNA ratio was not associated with incident plaque. Other factors that were significant in the multivariable models were Black race (IRR, 0.16 [95% CI, 0.03-0.99]; *P* = .049) and ART (IRR, 1.36 [95% CI, 1.01-1.82] *P* = .04) (eTables 3-5 in the Supplement). Removing CD4 from the models or substituting CD4:CD8 ratio or CD4 cell count nadir did not affect the association between the measures of viral persistence and incident plaque. There was no association between duration of ART, including protease inhibitor use or current abacavir or tenofovir use, and incident plaque.

**Table 4.** Association of Viral Persistence Measures With Risk of Incident Plaque Among Patients Without Plaque at Baseline^a^

Parameter	Unadjusted		Demographic adjusted^b^		Multivariable adjusted^c^		
	IRR (95% CI)	*P* value	IRR (95% CI)	*P* value	IRR (95% CI)	*P* value
RNA (per IQR)	3.05 (1.49-6.27)	.002	2.60 (1.24-5.47)	.01	4.05 (1.44-11.36)	.008
DNA (per IQR)	3.15 (1.51-6.57)	.002	2.97 (1.40-6.30)	.004	3.35 (1.22-9.19)	.02
RNA:DNA ratio, tertile 2 vs 1	1.42 (0.71-2.84)	.32	1.24 (0.63-2.43)	.53	1.82 (0.71-4.70)	.22
RNA:DNA ratio, tertile 3 vs 1	0.27 (0.07-1.07)	.06	0.29 (0.08-1.10)	.07	0.33 (0.05-2.35)	.27

Abbreviations: IQR, interquartile range; IRR, incidence rate ratio.

^a^ Includes 66 participants. Multiple imputation for missing values with 10 repetitions was applied.

^b^ Controlled for age, sex, and race.

^c^ Controlled for demographics (age, sex, and race), traditional cardiovascular risk factors (smoking, diabetes, hypertension, history of cardiovascular disease, low-density lipoprotein level, body mass index, use of medication to lower cholesterol level, and use of antihypertensives), HIV-related risk factors (duration of HIV infection, duration of use of antiretroviral therapy, and CD4:CD8 ratio), hepatitis C virus coinfection, and interleukin 6 level.

### Factors Associated With Higher Cell-Associated HIV RNA, DNA, and RNA:DNA Ratio

For our secondary analyses, we examined which factors (demographic, HIV-related, and inflammatory markers) were associated with higher levels of the viral persistence measures (Table 5). Cell-associated HIV RNA had a significant positive association with duration of HIV infection (difference, 7.7% [95% CI, 0.9%-15.0%]; *P* = .03) and C-reactive protein (difference, 20.7% [95% CI, 0.9%-44.4%] per doubling; *P* = .04) and a significant negative association with male sex (−89.7% [95% CI, −97.1% to −63.4%]; *P* < .001) and CD4:CD8 ratio (−44.7% [95% CI, −59.7% to −24.1%] per doubling; *P* < .001). Cell-associated HIV DNA was positively associated with sCD14 (18.6% [95% CI, 3.5%-35.8%] per 10% increase; *P* = .01) and negatively associated with CD4:CD8 ratio (−44.1% [95% CI, −59.9% to −22.0%] per doubling; *P* = .001). The HIV RNA:DNA ratio was positively associated with sCD163 (63.8% [95% CI, 3.5%-159.4%]; *P* = .04) but not any of the demographic or HIV characteristics.

**Table 5.** Factors Associated With Relative Differences in HIV RNA, HIV DNA, and RNA:DNA ratio^a^

Parameter	HIV RNA^b^		HIV DNA^b^		RNA/DNA ratio^b^	
	Estimate, % (95% CI)	*P* value	Estimate, % (95% CI)	*P* value	Estimate, % (95% CI)	*P* value
Age (per year)	2.7 (−1.7 to 7.3)	.23	3.4 (−1.2 to 8.1)	.15	0.6 (−3.7 to 5.0)	.79
Male	−89.7 (−97.1 to −63.4)	<.001	−68.0 (−91.4 to 19.4)	.09	−70.4 (−91.8 to 7.7)	.07
Black	−46.1 (−78.9 to 38.0)	.20	−33.2 (−74.8 to 77.1)	.427	−24.1 (−71.0 to 99.1)	.58
Other race	−10.9 (−65.2 to 128.2)	.81	139.5 (−13.1 to 559.9)	.09	−53.9 (−82.5 to 21.4)	.12
HIV duration (per year)	7.7 (0.9 to 15.0)	.03	NA	NA	NA	NA
CRP level (per doubling)	20.7 (0.9 to 44.4)	.04	NA	NA	NA	NA
CD4:CD8 ratio (per doubling)	−44.7 (−59.7 to −24.1)	<.001	−44.1 (−59.9 to −22.0)	.001	NA	NA
Soluble CD14 count (per 10% increase)	NA	NA	18.6 (3.5 to 35.8)	.01	NA	NA
Soluble CD163 count (per doubling)	NA	NA	NA	NA	63.8 (3.5 to 159.4)	.04

Abbreviations: CRP, C-reactive protein; NA, not applicable.

^a^ Includes 152 participants. A full-information maximum-likelihood approach was used for missing values.

^b^ All models are adjusted for demographics and additionally included HIV-related factors and markers of inflammation or coagulation that remained significant in the multivariable models.

## Discussion

Previous research has established that uncontrolled HIV disease is associated with increased cardiovascular risk. However, higher rates of myocardial infarction and atherosclerosis continue to be seen in PLWH in whom HIV is well controlled. Our study sought to explore this phenomenon further by evaluating cardiovascular risk in the setting of treated and suppressed HIV disease using more sensitive assessments of the viral load. In a cohort of PLWH with treated and suppressed disease, we found that levels of cell-associated HIV RNA and DNA were independently associated with incident carotid plaque. The association remained significant even after adjustment for traditional risk factors, HIV disease characteristics, and markers of inflammation, such as plasma IL-6 level. To our knowledge, our study is the first to demonstrate an association between the HIV reservoir and CVD in HIV. The independent association between cell-associated HIV RNA and DNA and the development of carotid plaque provides further evidence that HIV infection itself accelerates vascular disease. Although early initiation of ART reduced both AIDS-related and non–AIDS-related events in the START (Strategic Timing of Antiretroviral Treatment) study, the benefit of this strategy on cardiovascular events has not been established.^43^ Our findings suggest that therapies that can reduce the size of the reservoir, which could include early ART initiation or HIV curative strategies, may have a beneficial effect on reducing cardiovascular risk—a hypothesis that will need to be evaluated in future clinical trials.

Prior work has demonstrated that HIV infection is associated with higher carotid IMT^35,44,45^ and that measures of HIV disease severity, such as lower CD4 count nadir,^35^ were associated with higher carotid IMT. In addition to traditional risk factors, inflammatory markers—including high-sensitivity C-reactive protein,^32^ expression of certain monocyte markers,^46^ sCD163 and sCD14,^47,48^ CCR5,^33,49^ and plasma IL-6^33^—are associated with higher levels of vascular disease as assessed by carotid IMT. Our study adds to this literature by demonstrating an association between cell-associated HIV RNA and DNA and incident carotid plaque development in the setting of treated HIV disease.

Interestingly, we found that the measures of viral persistence were associated with incident plaque formation but not annual IMT progression. It is plausible that the underlying pathophysiology of plaque formation may be most susceptible to the inflammatory effects of HIV. Carotid plaque reflects different pathophysiology as opposed to IMT and represents intimal thickening, including foam cells, smooth muscle cells, macrophages, lipid core, and fibrous cap.^50^ Several studies^15,51,52^ have suggested that carotid plaque may be a more clinically significant measure. A meta-analysis of 16 studies including more than 56 000 individuals^15^ showed that mean IMT is significantly associated with cardiovascular events, but IMT progression over time was not. Although there is heterogeneity among studies regarding plaque definition and measurement, another meta-analysis of 11 population-based studies including more than 54 000 individuals^51,52^ demonstrated that carotid plaque compared with IMT had significantly higher accuracy for prediction of future cardiovascular events. In the general population, most studies demonstrate that IMT does not provide additional prognostic information compared with traditional risk calculators. In addition, different imaging modalities demonstrate that atherosclerosis in HIV infection is distinct from that of the general population. Persons living with HIV tend to have increased formation of noncalcified plaques that have thin fibroatheroma caps, making them more prone to rupture and CVD-related events,^12,13^ along with higher arterial inflammation that is related to inflammatory markers.^11,53^

We also found no association between viral persistence measures and baseline carotid IMT. We suspect that the very strong associations between atherosclerosis and traditional risk factors, such as smoking history, likely made it difficult to demonstrate more subtle associations between baseline IMT and viral persistence. Atherosclerosis is a chronic disease condition often requiring exposure to risk factors over an extended time interval, and accordingly, it can be challenging to extricate the cardiovascular implication of HIV infection. For this reason, we examined IMT progression and incident plaque development in addition to cross-sectional carotid IMT at baseline.

Among individuals with treated HIV infection, cell-associated HIV RNA is a possible indicator of residual virus production and perhaps replication and functions as a virologic biomarker to ascertain the effectiveness of curative therapies for HIV.^54,55,56,57^ Total DNA quantification is a highly sensitive ascertainment of the frequency of cells that are infected, but only a small and variable proportion of these cells have intact genomes capable of supporting virus.^23^ The ability to quantify the size of the reservoir in a sensitive and specific manner remains a major barrier in HIV research. Although some studies have reported an association between the frequency of cells that are HIV infected and measures of inflammation or immune dysfunction, few studies^58,59^ have examined the effect of viral persistence on overall morbidity. Our study is among the first to establish such a link for cardiovascular events. We found that IQR increases in RNA and DNA were associated with a 4.00-fold and 3.35-fold increased risk of incident plaque, respectively, after adjustment for traditional and HIV-related risk factors. These effect sizes are larger than what has been reported in other studies of traditional risk factors for carotid plaque. For example, in the Multi-Ethnic Study of Atherosclerosis, Tattersal et al^60^ reported an odds ratio of 1.6 per decade of aging and 2.3 for current smoking. These effect sizes are equivalent to a risk ratio of approximately 1.2 for aging and 1.3 for smoking, assuming a 56% rate of new plaque. Proving that the HIV reservoir is directly implicated in development of coronary artery disease will require interventions that effectively reduce or eliminate HIV-infected cells. Such interventions are not currently available, but multiple approaches are now being studied.

Chronic inflammation and immune dysfunction are closely linked to HIV persistence.^25,26,27,28,29^ For example, chronic inflammation and immune activation may lead to HIV persistence by a variety of mechanisms, including increasing production of virus, generation of new target cells, stimulating the proliferation of infected cells, and causing a chronic inflammatory environment that prevents the adaptive immune system from effectively eliminating infected cells.^61^ If this is the case, then strategies to reduce inflammation may affect the reservoir, as has been demonstrated in nonhuman primates.^62^ Our results suggest that decreasing the HIV reservoir size through strategies such as early ART might have an effect on reducing atherosclerosis and CVD risk in this population, although further research is needed. Although anecdotal and not directly related to our results, Sanchez et al^63^ previously observed that the degree of fibrosis in mucosal tissues was lower in the Berlin Patient—who was effectively cured via an allogenic stem cell transplant—then in elite controllers or people receiving long-term ART. This observation is broadly consistent with the hypothesis that HIV persistence has a detrimental effect on health.

### Limitations

Several limitations exist in this study. First, the short duration of follow-up may have limited our ability to demonstrate associations between the viral persistence markers and annual IMT progression. In addition, although IMT was measured at 2 time points, viral persistence parameters and inflammatory markers were only measured at baseline, so we cannot assess the effect of changes in these variables over time. The small sample size of our study also limited the number of candidate covariates that could be included in the multivariable models examining IMT outcomes. For example, among the inflammatory markers, only IL-6 was included as a candidate covariate owing to its previously demonstrated strong association with carotid IMT.^33^ The generalizability of this study among all PLWH, particularly in resource-limited settings, is unknown. This study assesses IMT and plaque in the carotid arteries and not a direct measurement of coronary artery plaque. Inflammation could be driving both viral persistence and carotid IMT, and further research is needed to elucidate these associations. We also noted that certain traditional risk factors, such as diabetes and use of medications to lower cholesterol levels, were surprisingly not associated with IMT in our models. This outcome may be owing to lack of specific data about the degree of diabetes control and adherence to medications to lower cholesterol levels in the cohort. In addition, about 20% of the study population was missing data on inflammatory markers, although in our analyses of the missing data, these data appeared to be missing at random and were imputed for the multivariable analyses. Finally, our measures of viral persistence have several important limitations. Most HIV DNA measured by quantitative polymerase chain reaction is defective and does not represent replication-competent virus,^23,64^ although defective provirus may contribute to continued inflammation and pathology in individuals receiving ART.^65^ In addition, HIV resides mainly in lymphoid tissue beyond the peripheral circulation,^66^ which would require more invasive sampling to access. Nevertheless, quantitative measures of HIV DNA and RNA from peripheral blood CD4^+^ T cells, as used in our study, remain important and are commonly used to quantify viral persistence. Future studies ought to assess the effects of intact vs nonintact cell-associated DNA and the effect of the lymphoid viral reservoir on cardiovascular outcomes.

## Conclusions

In the setting of well-controlled HIV infection, both cell-associated HIV RNA and DNA, which are measures of the HIV reservoir, were independently associated with incident carotid plaque, suggesting that viral persistence and ongoing subclinical viral expression underlie HIV-associated CVD. Strategies targeting the size of the reservoir as a means to cure or control HIV may help reduce the burden of comorbid disease in PLWH. Multiple strategies aimed at reservoir reduction are now being pursued in the clinic.

## Notes

Supplement. **eMethods.** Measuring Viral Persistence Parametmers **eTable 1.** Full Multivariable Model Examining Association of RNA:DNA Ratio With Annual Carotid IMT Progression (n = 152) **eTable 2.** Full Multivariable Model Examining Association of RNA:DNA Ratio With Annual Carotid IMT Progression, With CD4:CD8 Ratio Removed (n = 152) **eTable 3.** Full Multivariable Model Examining Association of HIV RNA With Incident Plaque (n = 152) **eTable 4.** Full Multivariable Model Examining Association of HIV DNA With Incident Plaque (n = 152) **eTable 5.** Full Multivariable Model Examining Association of RNA:DNA Ratio With Incident Plaque (n = 152)
