# Supplementary material for: Association of Viral Persistence and Atherosclerosis in Adults With Treated HIV Infection
Source: JAMA Netw Open. 2020 Oct 29;3(10):e2018099. doi: 10.1001/jamanetworkopen.2020.18099 (PMC7596582; doi:10.1001/jamanetworkopen.2020.18099)
Supplement: Supplement. — eMethods. Measuring Viral Persistence Parametmers eTable 1. Full Multivariable Model Examining Association of RNA:DNA Ratio With Annual Carotid IMT Progression (n = 152) eTable 2. Full Multivariable Model Examining Association of RNA:DNA Ratio With Annual Carotid IMT Progression, With CD4:CD8 Ratio Removed (n = 152) eTable 3. Full Multivariable Model Examining Association of HIV RNA With Incident Plaque (n = 152) eTable 4. Full Multivariable Model Examining Association of HIV DNA With Incident Plaque (n = 152) eTable 5. Full Multivariable Model Examining Association of RNA:DNA Ratio With Incident Plaque (n = 152) [file jamanetwopen-e2018099-s001.pdf]

Supplementary Online Content

McLaughlin MM, Ma Y, Scherzer R, et al. Association of viral persistence and atherosclerosis in adults with treated HIV infection. *JAMA Netw Open*. 2020;3(10):e2018099. doi:10.1001/jamanetworkopen.2020.18099

eMethods. Measuring Viral Persistence Parameters
eTable 1. Full Multivariable Model Examining Association of RNA:DNA Ratio With Annual Carotid IMT Progression (n = 152)
eTable 2. Full Multivariable Model Examining Association of RNA:DNA Ratio With Annual Carotid IMT Progression, With CD4:CD8 Ratio Removed (n = 152)
eTable 3. Full Multivariable Model Examining Association of HIV RNA With Incident Plaque (n = 152)
eTable 4. Full Multivariable Model Examining Association of HIV DNA With Incident Plaque (n = 152)
eTable 5. Full Multivariable Model Examining Association of RNA:DNA Ratio With Incident Plaque (n = 152)

This supplementary material has been provided by the authors to give readers additional information about their work.

©2020 McLaughlin MM et al. *JAMA Network Open*.

eMethods. Measuring Viral Persistence Parameters

Cryopreserved PBMCs were rapidly thawed and $CD4^+$ T cells were enriched to high purities by negative selection using the EasySep Human $CD4^+$ T cell enrichment kit (Stemcell Technologies, Vancouver, British Columbia, Canada). Cellular RNA and DNA from enriched CD4+ T cells were purified using the AllPrep DNA/RNA kit (Qiagen, Ventura CA) as specified by the manufacturer, quantified using a Nanodrop (ND-1000) spectrophotometer and normalized to cell equivalents by qPCR using human genomic TERT for DNA and RPLP0 expression for RNA (Life Technologies, Grand Island NY). Total cellular HIV-1 DNA and HIV-1 RNA (unspliced and multiply spliced) was quantified with a qPCR TaqMan assay using LTR-specific primers F522-43 (5' GCC TCA ATA AAG CTT GCC TTG A 3'; HXB2 522–543) and R626-43 (5' GGG CGC CAC TGC TAG AGA 3'; 626–643) coupled with a FAM-BQ probe (5' CCA GAG TCA CAC AAC AGA CGG GCA CA 3)[66] on a StepOne Plus Real-time PCR System (Applied Biosystems Inc, Foster City CA). Cell-associated HIV-1 RNA copy number was determined in a reaction volume of 20 µl containing 10 µl of 2x TaqMan RNA-to-Ct One Step kit (Life Technologies), 4 pmol of each primer, 4pmol of probe, 0.5 µl reverse transcriptase, and 5µl of RNA under identical cycling conditions. Cycling conditions were 48°C for 20 min, 95°C for 10 min, then 60 cycles of 95°C for 15s and 59 °C for 1 min. RPLP0 copy number was determined under the same cycling conditions however only 40 cycles were performed. Cell associated HIV-1 DNA copy number was determined using a reaction volume of 20 µl with 10 µl of 2x TaqMan Universal Master Mix II including UNG (Life technologies), 4 pmol of each primer, 4 pmol of probe, and 5 µl of DNA. Cycling conditions were 50°C for 2 min, 95°C for 10 min, then 60 cycles of 95°C for 15s and 59°C for 1 min. TERT copy number was similarly determined however extension was performed at 60°C and only 40 cycles were performed. For HIV-1 DNA measurements, external quantitation standards were prepared from pNL4-3 in a background of HIV-1 negative human cellular DNA, calibrated to the Virology Quality Assurance (VQA, NIH Division of AIDS) cellular

©2020 McLaughlin MM et al. *JAMA Network Open*.

DNA quantitation standards. For HIV-1 RNA measurements, external quantitation standards were prepared from full length NL4-3 virion RNA followed by copy number determination using the Abbott RealTime assay (Abbott Diagnostics, Des Plains Ill) and calibrated to VQA HIV-1 RNA standards. Patient specimens were assayed with up to 800 ng total cellular RNA or DNA in replicate reaction wells and copy number determined by extrapolation against a 7-point standard curve (1–10,000 cps) performed in triplicate.

©2020 McLaughlin MM et al. *JAMA Network Open*.

eTable 1. Full Multivariable Model Examining Association of RNA:DNA Ratio With Annual Carotid IMT Progression (n = 152)[a,b]

Parameter	Annual CIMT progression in mm (95%CI)	p-value
RNA/DNA tertile 2	-0.029 (-0.061, 0.002)	0.071
RNA/DNA tertile 3	-0.030 (-0.061, 0.002)	0.062
Age (years)	0.003 (0.001, 0.005)	0.002
Male	-0.072 (-0.125, -0.019)	0.008
Black	-0.010 (-0.047, 0.028)	0.619
Other race/ethnicity	0.021 (-0.016, 0.059)	0.262
Smoking (per 10 pack-years)	0.003 (-0.006, 0.012)	0.514
Diabetes	-0.014 (-0.063, 0.035)	0.580
Hypertension	0.063 (0.012, 0.113)	0.015
Cardiovascular disease	0.042 (-0.010, 0.094)	0.116
LDL (per doubling)	-0.014 (-0.041, 0.013)	0.309
BMI (per 5 kg/m^2)	-0.007 (-0.020, 0.006)	0.289
Cholesterol medications	-0.003 (-0.036, 0.029)	0.835
Hypertension medications	-0.076 (-0.131, -0.022)	0.006
HIV duration	0.0012 (-0.002, 0.004)	0.432
Antiretroviral therapy	0.002 (-0.003, 0.007)	0.333
CD4:CD8 ratio (per doubling)	-0.007 (-0.021, 0.006)	0.284
Hepatitis C	-0.004 (-0.038, 0.030)	0.818
IL-6 (per doubling)	0.005 (-0.009, 0.019)	0.481

[a] Multivariable adjusted models control for demographics (age, sex, race), traditional CVD risk factors (smoking, diabetes, hypertension, history of CVD, LDL, BMI, use of cholesterol lowering medication, use of anti-hypertensive), and HIV-related related risk factors (duration of HIV infection, duration of ART, CD4:CD8), hepatitis C, and IL-6
[b] Full information maximum likelihood approach was used for missing values

©2020 McLaughlin MM et al. *JAMA Network Open*.

eTable 2. Full Multivariable Model Examining Association of RNA:DNA Ratio With Annual Carotid IMT Progression, With CD4:CD8 Ratio Removed (n = 152)[a,b]

Parameter	Annual CIMT progression in mm (95%CI)	p-value
RNA/DNA tertile 2	-0.032 (-0.063, -0.001)	0.045
RNA/DNA tertile 3	-0.032 (-0.063, -0.001)	0.044
Age (years)	0.003 (0.001, 0.005)	0.003
Male	-0.068 (-0.121, -0.015)	0.012
Black	-0.01 (-0.048, 0.028)	0.602
Other race/ethnicity	0.022(-0.015, 0.060)	0.239
Smoking (per 10 pack-years)	0.004 (-0.005, 0.013)	0.381
Diabetes	-0.011 (-0.061, 0.038)	0.653
Hypertension	0.064 (0.013, 0.114)	0.013
Cardiovascular disease	0.041 (-0.012, 0.093)	0.128
LDL (per doubling)	-0.015 (-0.043, 0.012)	0.268
BMI (per 5 kg/m^2)	-0.007 (-0.020, 0.006)	0.296
Cholesterol medications	-0.006 (-0.038, 0.026)	0.712
Hypertension medications	-0.076 (-0.131, -0.022)	0.006
HIV duration	0.0016(-0.001, 0.004)	0.280
Antiretroviral therapy	0.002(-0.003, 0.007)	0.411
Hepatitis C	-0.004(-0.038, 0.030)	0.813
IL-6 (per doubling)	0.006(-0.008, 0.020)	0.426

[a] Multivariable adjusted models control for demographics (age, sex, race), traditional CVD risk factors (smoking, diabetes, hypertension, history of CVD, LDL, BMI, use of cholesterol lowering medication, use of anti-hypertensive), and HIV-related related risk factors (duration of HIV infection, duration of ART), hepatitis C, and IL-6. CD4:CD8 ratio was removed.
[b] Full information maximum likelihood approach was used for missing values

©2020 McLaughlin MM et al. *JAMA Network Open*.

eTable 3. Full Multivariable Model Examining Association of HIV RNA With Incident Plaque (n = 152)[a,b]

Parameter	Incident risk ratio (95%CI)	p-value
RNA (per doubling)	1.50 (1.12, 2.00)	0.006
Age (years)	1.00 (0.92, 1.10)	0.954
Male	1.81 (0.11, 29.15)	0.668
Black	0.16 (0.03, 0.99)	0.049
Other race/ethnicity	0.54 (0.15, 1.89)	0.332
Smoking (per 10 pack-years)	1.27 (0.89, 1.81)	0.190
Diabetes	0.19 (0.02, 2.04)	0.171
Hypertension	0.24 (0.02, 2.52)	0.231
Cardiovascular disease	1.72 (0.24, 12.41)	0.588
LDL (per doubling)	0.68 0.27, 1.71)	0.412
BMI (per 5 kg/m^2)	1.34 (0.81, 2.21)	0.260
Cholesterol medications	0.98 (0.14, 6.96)	0.981
Hypertension medications	2.88 (0.26, 32.45)	0.382
HIV duration	0.98 (0.82, 1.17)	0.825
Antiretroviral therapy	1.36 (1.01, 1.82)	0.040
CD4:CD8 ratio (per doubling)	1.24 (0.82, 1.88)	0.297
Hepatitis C	0.42 (0.14, 1.29)	0.130
IL-6 (per doubling)	0.66 (0.40, 1.08)	0.095

[a] Multivariable adjusted models control for age, sex, race, traditional CVD risk factors (smoking, diabetes, hypertension, history of CVD, LDL, BMI, use of cholesterol lowering medication, use of anti-hypertensive), and HIV-related related risk factors (duration of HIV infection, duration of ART, CD4:CD8), hepatitis C, and IL-6

[b] Multiple imputation for missing values with the Markov Chain Monte Carlo method was used

©2020 McLaughlin MM et al. *JAMA Network Open*.

eTable 4. Full Multivariable Model Examining Association of HIV DNA With Incident Plaque (n = 152)[a,b]

Parameter	Incident risk ratio (95%CI)	p-value
DNA (per doubling)	1.29 (1.04, 1.61)	0.022
Age (years)	1.01 (0.94, 1.09)	0.758
Male	0.78 (0.17, 3.58)	0.746
Black	0.59 (0.19, 1.80)	0.351
Other race/ethnicity	1.14 (0.73, 1.79)	0.568
Smoking (per 10 pack-years)	0.30 (0.05, 2.06)	0.222
Diabetes	0.78 (0.32, 1.89)	0.579
Hypertension	0.93 (0.62, 1.39)	0.724
Cardiovascular disease	0.78 (0.18, 3.49)	0.748
LDL (per doubling)	1.37 (0.61, 3.10)	0.452
BMI (per 5 kg/m^2)	1.09 (0.88, 1.35)	0.442
Cholesterol medications	1.19 (0.98, 1.45)	0.077
Hypertension medications	1.07 (0.76, 1.50)	0.689
HIV duration	0.85 (0.31, 2.33)	0.747
Antiretroviral therapy	0.78 (0.51, 1.17)	0.227
CD4:CD8 ratio (per doubling)	1.29 (1.04, 1.61)	0.022
Hepatitis C	1.011 (0.941, 1.087)	0.758
IL-6 (per doubling)	0.776 (0.168, 3.584)	0.746

[a] Multivariable adjusted models control for age, sex, race, traditional CVD risk factors (smoking, diabetes, hypertension, history of CVD, LDL, BMI, use of cholesterol lowering medication, use of anti-hypertensive), and HIV-related related risk factors (duration of HIV infection, duration of ART, CD4:CD8), hepatitis C, and IL-6
[b] Multiple imputation for missing values with the Markov Chain Monte Carlo method was used

©2020 McLaughlin MM et al. *JAMA Network Open*.

eTable 5. Full Multivariable Model Examining Association of RNA:DNA Ratio With Incident Plaque (n = 152)[a,b]

Parameter	Incident risk ratio (95%CI)	p-value
RNA/DNA tertile 2	1.43 (0.54, 3.73)	0.471
RNA/DNA tertile 3	0.29 (0.04, 2.26)	0.239
Age (years)	1.03 (0.95, 1.10)	0.516
Male	0.53 (0.19, 1.47)	0.224
Black	1.12 (0.75, 1.69)	0.582
Other race/ethnicity	0.51 (0.09, 2.89)	0.449
Smoking (per 10 pack-years)	0.86 (0.37, 2.00)	0.725
Diabetes	0.78 (0.50, 1.23)	0.289
Hypertension	0.51 (0.11, 2.28)	0.376
Cardiovascular disease	1.73 (0.53, 5.61)	0.362
LDL (per doubling)	1.08 (0.89, 1.32)	0.428
BMI (per 5 kg/m^2)	1.11 (0.94, 1.32)	0.228
Cholesterol medications	0.94 (0.69, 1.27)	0.664
Hypertension medications	0.87 (0.27, 2.82)	0.816
HIV duration	0.62 (0.38, 0.98)	0.042
Antiretroviral therapy	1.43 (0.54, 3.73)	0.471
CD4:CD8 ratio (per doubling)	0.29 (0.04, 2.26)	0.239
Hepatitis C	1.03 (0.95, 1.10)	0.516
IL-6 (per doubling)	0.53 (0.19, 1.47)	0.224

[a] Multivariable adjusted models control for age, sex, race, traditional CVD risk factors (smoking, diabetes, hypertension, history of CVD, LDL, BMI, use of cholesterol lowering medication, use of anti-hypertensive), and HIV-related related risk factors (duration of HIV infection, duration of ART, CD4:CD8), hepatitis C, and IL-6

[b] Multiple imputation for missing values with the Markov Chain Monte Carlo method was used

©2020 McLaughlin MM et al. *JAMA Network Open*.
